# Longitudinal Study on the Progression of Diabetes Mellitus Patients at Jimma University Specialized Hospital, Ethiopia

**DOI:** 10.1155/jdr/7035393

**Published:** 2025-12-03

**Authors:** Kindu Kebede Gebre, Million Wesenu Demissie, Habtamu Abebe Getahun, Assefa Legesse Sisay

**Affiliations:** ^1^Department of Statistics, College of Computing and Informatics, Haramaya University, Dire Dawa, Ethiopia; ^2^Department of Epidemiology, Public Health Faculty, Institute of Health, University of Gondar, Gondar, Ethiopia; ^3^Departments of Epidemiology, Faculty of Public Health, Jimma University, Jimma, Ethiopia

**Keywords:** diabetes mellitus, fasting blood sugar, growth curve analysis, multilevel model

## Abstract

**Background:**

Diabetes mellitus is a metabolic disorder marked by elevated blood sugar levels. This study is aimed at identifying the factors influencing fasting blood sugar levels and at assessing treatment changes both within and across patients.

**Methods:**

A retrospective cohort study design was employed to collect relevant data from 100 patients with diabetes, comprising 861 repeated measurements, aged 18 years and above, between September 11, 2018, and October 11, 2021. We utilized a multilevel random coefficient model with time-varying covariates to identify determinants and growth curve analysis to describe patterns of change over time. Additionally, pairwise least square means differences were analyzed to evaluate treatment effects during the follow-up period.

**Results:**

The variability in fasting blood sugar levels among patients was 29.8%, while 70.2% of the variability was attributed to changes within individual patients. Significant associations were found between fasting blood sugar levels and pulse rate, high-density lipoprotein cholesterol levels, and baseline fasting blood sugar levels.

**Conclusion:**

The findings indicate that fasting blood sugar levels in patients increase as pulse rate, high-density lipoprotein levels, and baseline fasting blood sugar levels rise, with statistical significance at the 5% alpha level. Therefore, it is crucial to monitor these factors closely during patient follow-ups to optimize management strategies.

## 1. Background

The World Health Organization (WHO) describes diabetes mellitus as a chronic illness characterized by high blood sugar levels and associated multisystem complications. This condition results from defects in insulin secretion or action, leading to ineffective glucose transport from the bloodstream. Diabetes is a prevalent and chronic disease that requires effective self-management by patients [1]. The rising rates of obesity and overweight are significantly contributing to the increasing burden of diabetes mellitus and other chronic health issues. A person is diagnosed with diabetes when their fasting blood sugar (FBS) level is equal to or greater than 7.0 mmol/L (126 mg/dL), their 2 h blood sugar level is equal to or greater than 11.1 mmol/L (200 mg/dL), or their glycated hemoglobin (HbA1c) level is 6.5% or higher [2]. Diabetes disrupts multiple metabolic pathways, leading to abnormalities in fat, protein, and carbohydrate metabolism caused by defects in insulin secretion and insulin resistance, resulting in chronic hyperglycemia [3]. Failure to treat diabetes properly can result in serious health complications, such as visual impairment and nerve damage [4–7]. As emphasized by Thorne et al. [8], individuals with diabetes must manage their food intake, monitor their blood glucose levels throughout the day, and may require medication during social activities.

Diabetes can be categorized into two main types based on its pathophysiology: Type I and Type II diabetes mellitus. Type I diabetes is caused by autoimmune destruction of pancreatic *β*-cells, resulting in loss of insulin secretion, while Type II diabetes primarily stems from insulin resistance, affecting how the body utilizes its endogenous insulin [8]. In Type II diabetes, patients often retain some insulin production, and the incidence of ketoacidosis is significantly lower than in Type I, where ketoacidosis can occur under stress from illness, such as infection [9]. The WHO classifies diabetes into three categories: Type I diabetes, Type II diabetes, and gestational diabetes. Type I diabetes results from autoimmune attacks against the insulin-producing *β*-cells, leading to an inability to produce necessary insulin. In contrast, Type II diabetes involves the body producing insulin that is rendered ineffective due to resistance, leading to high blood sugar levels due to insufficient insulin action or production. Gestational diabetes occurs during pregnancy and poses significant health risks for both mother and child [10].

Research has identified various risk factors for Type I diabetes, including family history, infections, viral illnesses, the presence of harmful immune system cells, and dietary deficiencies such as low vitamin D intake [11]. For Type II diabetes, risk factors include excess body weight, physical inactivity, poor nutrition, family history, previous gestational diabetes, and older age [12]. According to a report from the WHO [13], factors like overweight status, family history of diabetes, age, type of diabetes, blood pressure, and gender significantly impact FBS levels. A study conducted in India identified genetic factors, tobacco use, limited physical activity, and systolic hypertension as strong risk factors [14]. Another study in Uganda found a prevalence of 18.7% for Type II diabetes among participants, with significant associations to age, alcohol use, smoking, body mass index (BMI), and family history [15]. In southern Ethiopia, waist circumference, BMI, smoking, hypertension, and total cholesterol levels were strongly linked to diabetes with a prevalence of 6.5% [16]. A mixed-effects longitudinal regression model appeared suitable for diabetes datasets, indicating that treatment duration, weight, educational level, and blood pressures significantly impact FBS levels in Ghana [17].

Globally, diabetes is a leading health problem, causing 1.6 million deaths among adults in 2016, with half a billion individuals affected [18]. The International Diabetes Federation (IDF) estimated that 415 million adults aged 20–79 were living with diabetes, including 193 million who were undiagnosed [19]. The increase in diabetes prevalence represents a growing health challenge, particularly in sub-Saharan African countries, alongside other noncommunicable diseases [20]. In Africa, the number of individuals living with diabetes was estimated at 12.1 million in 2010, projected to rise to 23.9 million by 2030 [21]. The economic burden of diabetes is also considerable, with an estimated cost of $2.1 trillion (2.2% of global GDP) by 2030, contributing to early mortality [18].

In Ethiopia, the prevalence of diabetes mellitus has shown an increasing trend, with recent national estimates ranging from 3.2% to 6.5%, particularly in urban areas with limited access to continuous care [2, 16]. These challenges underscore the need for context-specific, data-driven strategies for monitoring disease progression.

Although fasting blood glucose levels have been extensively studied, this work advances current knowledge by applying a multilevel longitudinal modeling approach that captures both within-patient changes and between-patient differences over time, offering more precise insights into glycemic control dynamics among diabetic patients. The study included 861 adult diabetic patients from Jimma University Specialized Hospital (JUSH), with up to 13 repeated FBS measurements per patient.

Several previous studies in Ethiopia have investigated diabetes prevalence using binary logistic regression models [22]. However, these models assume independence between observations, which is not appropriate for longitudinal data where repeated measurements on the same individual tend to be correlated. Longitudinal studies have the distinct advantage of tracking individual changes in outcome variables over time and associating these changes with other clinical factors.

For analyzing longitudinal data, parametric approaches such as linear mixed models and semiparametric mixed models are commonly used. While linear mixed models are efficient when correctly specified, they may impose overly restrictive assumptions on the mean trajectory of FBS. Semiparametric mixed models provide greater flexibility and are more effective in handling missing data typical in longitudinal studies.

This study utilized a multilevel random coefficient model with time-varying covariates to analyze FBS trajectories. The hierarchical structure of the data is as follows:

• Level 1 (within-patient level): Repeated FBS measurements over time (up to 13 visits per patient), capturing individual temporal trends and time-varying factors.
• Level 2 (between-patient level): Individual patients (*N* = 861), capturing differences in baseline characteristics and allowing random effects to reflect heterogeneity in glycemic progression.

By modeling both levels simultaneously, this approach provides a robust framework for understanding glycemic control patterns in a real-world Ethiopian clinical setting, overcoming limitations of prior cross-sectional or simpler longitudinal analyses.

## 2. Research Methodology

### 2.1. Study Design, Population, and Area

This study employed a retrospective cohort design to gather relevant information from antiretroviral therapy (ART) charts to fulfill its objectives. The participants included diabetes-positive patients aged 18 years and older who began ART on or after September 11, 2018, and who had baseline data along with at least two follow-up assessments until October 11, 2021. The research was conducted at JUSH, located in the South-Western Oromia Region of Ethiopia. The study population comprised adult diabetes-positive patients receiving ART at this hospital.

### 2.2. Data Source and Collection Procedures

All diabetes mellitus patients who were placed on insulin and attended follow-up visits from September 11, 2018, to October 11, 2021, at JUSH were included in this study. The focus was on adult diabetes-positive patients during the specified timeframe. Data were obtained from a retrospective cohort study based on an electronic ART database and a review of patient charts, which contained sociodemographic, laboratory, and clinical information for all diabetes patients under ART follow-up, including detailed follow-up histories from the hospital.

FBS levels were recorded at the initiation of treatment and at various subsequent time points. Patients visited the clinic regularly (approximately every 3 months), during which their FBS levels were measured and documented on individual follow-up cards.

### 2.3. Variables Included in the Study

The primary response variable analyzed in this study was the repeated FBS level, measured in milligrams per deciliter at each patient visit. Fixed sociodemographic and clinical factors anticipated to be associated with FBS levels in diabetic patients are outlined in Table 1.

### 2.4. Data Quality and Management of Missing Data

Data quality was ensured by assigning data collectors from the hospital's ART section, which underwent rigorous training provided by the Ethiopian Ministry of Health. One of the significant challenges in longitudinal studies is the presence of missing data. However, multilevel analysis offers flexibility in handling such missing data effectively [23].

### 2.5. Inclusion and Exclusion Criteria

#### 2.5.1. Inclusion Criteria

Patients aged 18 years and older who attended a minimum of two follow-up visits for treatment at the ART clinic and who began ART between September 11, 2018, and October 11, 2021, at Jimma University Hospital, were included in this study.

#### 2.5.2. Exclusion Criteria

Patients under 18 years of age attending the ART clinic for prescription refills, those not registered in the ART clinic, and those who had not initiated ART were excluded from this study. Additionally, patients whose treatment fell outside the specified study period were not included.

### 2.6. Statistical Data Analysis

Longitudinal studies are characterized by repeated measurements of the outcome variable, meaning that the same individuals are assessed multiple times. In such studies, the observations from a single individual over time are not independent of one another. Therefore, it is essential to employ specialized statistical techniques that account for the correlation of repeated observations within individuals.

To address the correlated nature of repeated measurements, corrections must be made for intrasubject correlations. This is done by assuming a specific working correlation structure for the repeated measurements. Various correlation structures may be selected, including independent, exchangeable, autoregressive, and unstructured correlation structures. It is crucial to identify the appropriate working correlation structure for the outcome variable's repeated measurements.

In the context of a hierarchical or nested data structure where individuals are grouped within organizational units, or where multiple measurements are taken from the same individual, multilevel analysis is employed. These methods allow exploring relationships between variables measured at different levels of the data hierarchy. It represents a generalization of ordinary least squares regression analysis, accommodating the complexities of estimating models with two or more levels. The fundamental statistical model in multilevel analysis involves successive sampling from each level of a hierarchical population.

Before conducting a multilevel model analysis, the study must make key decisions, including which predictors to include and whether the parameter values will be fixed, random, or a combination of both.

#### 2.6.1. Intraclass Correlation Coefficient (ICC)

The first step in multilevel analysis is to define a coefficient that indicates the proportion of variance in the outcome variable attributed to variation between the higher level units. This coefficient is known as the ICC, also referred to as the variance partition coefficient (VPC), and is denoted by the Greek letter rho.

 $$\begin{array}{c} \rho =\frac{{\sigma^{2}}_{u0}}{{\sigma^{2}}_{u0}+{\sigma^{2}}_{e}}, \end{array}$$

where *σ*^2^_*u*0_ is the variance of the Level 2 or higher level residuals and *σ*^2^_*e*_ is the variance of the Level 1 or lowest level residuals. The variance of *e*_*ij*_ is denoted as *σ*^2^_*e*_ and the variance of *U*_0*j*_ as *σ*^2^_*u*0_. The percentage of observed variation in the dependent variable attributable to class-level characteristics is obtained by dividing *σ*^2^_*u*0_ by the total variance. *σ*^2^_*e*_ is the variance of the lowest level errors *e*_*ij*_, and *σ*^2^_*u*0_ is the variance of the highest level errors *u*_0*j*_.

The ICC is commonly utilized as a baseline for estimating variances at two levels that can be explained by more complex models. It serves to evaluate whether the variation at Level 2 is negligible. Although it is difficult to establish a universal threshold, a hierarchical data structure should not be disregarded if the ICC is 0.05 or higher. If the ICC is lower, it may be appropriate to consider a one-level model, potentially using robust standard error estimation.

#### 2.6.2. Growth Curve Analysis

Growth curve analysis is a statistical method used to describe patterns of change over time and determine the frequency of visits. Specifically, this approach allows for the estimation of the best fit line or curve for each individual's responses over time [24].

#### 2.6.3. Random Intercept Model

A random intercept model allows the intercepts to vary, meaning that the scores on the dependent variable for each individual observation are predicted by an intercept that differs across groups. This model assumes fixed slopes (which are the same across different contexts).

#### 2.6.4. Random Slope Model

In a random slope model, the slopes are allowed to vary, resulting in different slopes across groups. Conversely, this model assumes fixed intercepts (which are the same across different contexts).

#### 2.6.5. Random Intercept and Slope Model

A model that incorporates both random intercepts and random slopes is considered to be the most realistic, though it is also the most complex. In this model, both intercepts and slopes vary across groups, indicating that they differ in different contexts. This means that each group has its own regression model with distinct intercepts and slopes. As groups are sampled, the model assumes that the intercepts and slopes are randomly sampled from a population of group intercepts and slopes.

### 2.7. Variable Selection for Multilevel Analysis

For this study, forward variable selection was employed to choose explanatory variables. The initial step in this selection process involved fitting a univariate multilevel model for each covariate and evaluating it with an alpha level of 0.25.

### 2.8. Goodness of Fit Test

After developing a model using various techniques to estimate the model parameters, several mechanisms are used to assess its appropriateness, adequacy, and usefulness. First, the *t*-test statistic is commonly employed to evaluate the significance of individual regression coefficients for each independent variable. Second, a deviance-based test, or likelihood ratio test, serves as a general principle for assessing fixed multiparameter models and evaluating the random components of hierarchical linear models.

## 3. Results

### 3.1. Descriptive Statistics

This retrospective study analyzed 100 patients with repeated FBS measurements taken at follow-up visits every 3 months. Patients enrolled in the ART program at JUSH between September 11, 2018, and October 11, 2021. Each subject had between 3 and 13 observations. The descriptive statistics summarize FBS within each category level of key categorical covariates.

Among the participants, 365 (42.4%) were female, with an average FBS level of 170.17 mg/dL and a standard deviation of 71.34 mg/dL. The remaining 496 (57.6%) were male, presenting a mean FBS of 182.51 mg/dL (SD = 78.92 mg/dL), as shown in Table 2. A greater proportion of patients who visited for FBS measurement resided in rural areas (454 or 52.7%) and had a history of hypertension (475 or 55.2%). Notably, 55.2% of the diabetes patients whose FBS levels were documented also had hypertension, while 52.7% were from rural backgrounds. Additional details regarding categorical variables such as complications, age groups, and types of complications are presented in Table 2.

The summary of FBS levels at various time points is presented in Table 3. The data shows that the mean FBS levels decreased over time until the 18th month, followed by an increase after the 21st month, and then a decrease starting at the 33rd month. In contrast, the standard deviation of FBS levels did not exhibit a consistent pattern. It increased until the 6th month, then decreased from the 9th month through the 15th month, before rising again after the 18th month and declining at the 27th month. Additionally, the number of patients decreased as follow-up time progressed from the baseline to the 13th measurement visit, indicating intermittent missing data in the study.

Clinical and demographic information about the patients was gathered for this study at the start of ART and at several follow-up appointments during the observation period. Descriptive statistics for a number of important continuous clinical covariates that are measured repeatedly over time, such as weight, systolic and diastolic blood pressure, pulse rate, creatinine, urea, cholesterol (high-density lipoprotein [HDL] and low-density lipoprotein [LDL]), glucose, and triglycerides, are summarized in Table 4. Since these covariates were gathered at every planned visit and demonstrated significant changes throughout the follow-up period, they are time variant.

Many of these variables showed distinct temporal trends, according to the analysis. For instance, pulse rate increased gradually over time, reaching a peak at the 11th visit (30th month), which may indicate changes in treatment effects or cardiovascular response. Up until the 18th month, the patient's weight increased steadily. It then stabilized between the 21st and 33rd months, and then, it increased again, possibly as a result of metabolic changes brought on by ART or nutritional recovery. A delayed metabolic adaptation or intervention effect may be the cause of the glucose levels' steady rise until the eighth visit and subsequent decline through the 11th. Creatinine, urea, blood pressure, and lipid profiles (HDL, LDL, and total cholesterol) all showed comparable oscillations over time, reflecting the dynamic physiological reactions of ART-treated patients.

Overall, by showing the temporal patterns and variability of these clinical markers, Table 4 offers crucial context for understanding the longitudinal analysis. This emphasizes that rather than treating these covariates as static baseline characteristics, modeling efforts should treat them as time-dependent. Thus, their inclusion is warranted and essential for a thorough comprehension of patient outcomes over time.

### 3.2. Exploratory Data Analysis

**Figure 1:**
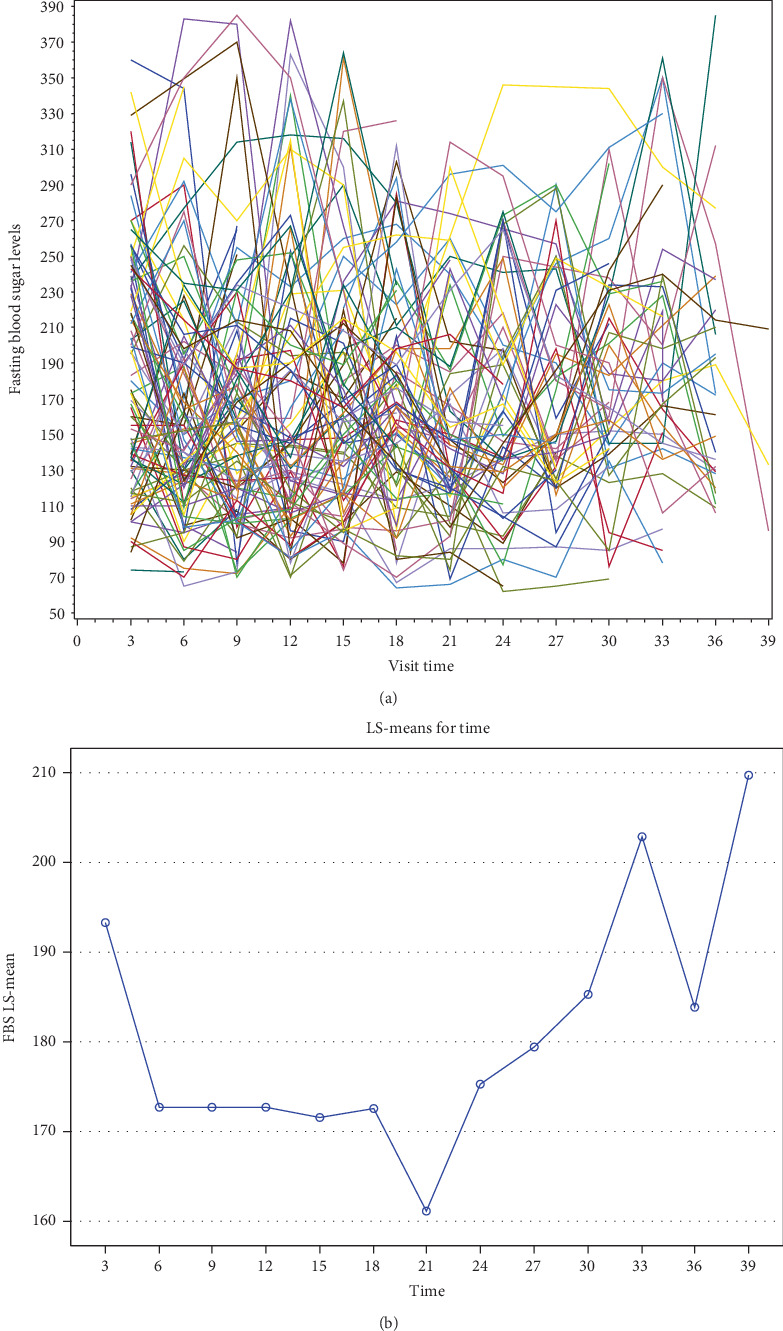
(a) Profile plot of fasting blood sugar levels for individual patients. (b) Mean–mean scatter plot over time for diabetes patients.

Individual FBS trajectory plots, also known as spaghetti plots, are shown in Figure 1a for each follow-up visit. Significant variation in FBS trends among patients is depicted in the plot; some patients' levels fluctuate over time, while others show a consistent rise. This pattern highlights the significance of using mixed-effects models to account for such variation by indicating significant heterogeneity in the intercepts and slopes of individual trajectories.

However, the spaghetti plot lacks adequate visual clarity because of the thick overlay of individual lines. It could be difficult to identify particular patterns or subgroup behaviors as a result. Future visualizations should emphasize specific trajectories or group-level summaries (e.g., by baseline FBS categories or by trend direction) to improve interpretability. Furthermore, a nonsystematic, scattered pattern is revealed by the mean-over-time scatter plot of arithmetic means across visits, underscoring the significant individual variability (refer to Figure 1 and see Supporting Information for the full SAS code).

**Figure 2:**
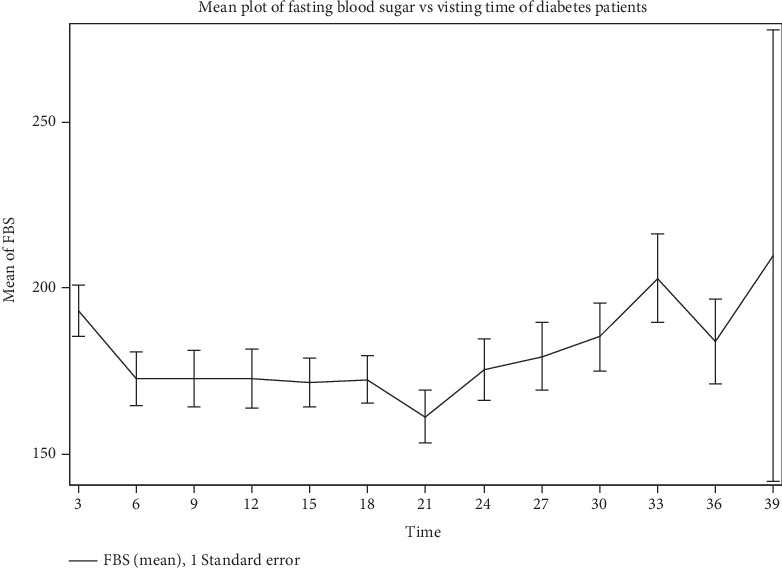
Mean plot of fasting blood sugar levels over visiting times for diabetes patients.

The mean plot of FBS levels over follow-up visits is shown in Figure 2, providing information on temporal trends and within-patient variability during the study period. According to the plot, some patients' FBS levels first decreased following their first visit, while others' levels remained largely stable until around the 18th visit. Between the 30th and 33rd visits, there is a discernible upward trend in mean FBS levels, which may indicate a late-phase rise in blood sugar levels during the follow-up period. This pattern demonstrates how FBS levels change over time and bolsters the argument for time-varying modeling techniques.

### 3.3. Statistical Analysis Using Intraclass Correlation

The study utilized the ICC to assess whether observations within diabetes patients are more similar to each other than to observations from different patients. An ICC of zero indicates perfect independence of residuals, meaning that the observations do not depend on patient membership. In such cases, simpler analytical techniques may be employed. Conversely, if the ICC approaches one, it suggests that there is no individual-level variance; everyone is the same. Understanding how much of the overall variation in the response can be attributed to differences between patients may be theoretically informative. Additionally, observing changes in the ICC as variables are included in the model can provide valuable insights to examining both between- and within-patient variances.

As shown in Table 5, the ICC indicates strong evidence of variability among patients. The intercept-only model estimates the intercept at 176.05, representing the average FBS level across all patients for each individual measurement. The variance of the individual observation residual errors, denoted as *σ*^2^_*e*_, is estimated at 4096.34. Meanwhile, the variance of the patient-level residual errors, represented by *σ*^2^_*u*0_, is estimated at 1739.84, both of which are statistically significant with a *p* value of less than 0.0001.

The ICC for this model is equal to *ρ* = *σ*^2^_*u*0_/(*σ*^2^_*u*0_ + *σ*^2^_*e*_) = 1739.84/(1739.84 + 4096.34) = 1739.84/5836.18 = 0.298.

Thus, 29.8% of the variation in FBS levels is attributed to differences between patients, while the remaining 70.2% of the variation occurs within patients, resulting in a deviance value of 9753.5.

Candidate variable selection was performed using univariate multilevel models for each covariate, evaluated at a significance level of 0.25. The results from these univariate models indicated that visiting time, pulse rate, hypertension status, age group, creatinine levels, LDL levels, HDL levels, triglycerides, baseline FBS, and cholesterol levels were all associated with FBS levels at this 25% significance level.

The results of the least squares mean comparisons of diabetic patients' FBS levels at various visit times are shown in Table 6. Every time point's *t*-test reveals highly significant differences (all *p* < 0.0001), suggesting that FBS levels alter significantly over the course of the follow-up.

The main goal of the study was to investigate how FBS levels change over time in patients undergoing treatment, and these results amply support that goal. Diabetes patients' dynamic glucose regulation during the course of treatment or monitoring is highlighted by the statistical significance at all measured time points, which indicates that time has a quantifiable and consistent impact on FBS levels.

**Figure 3:**
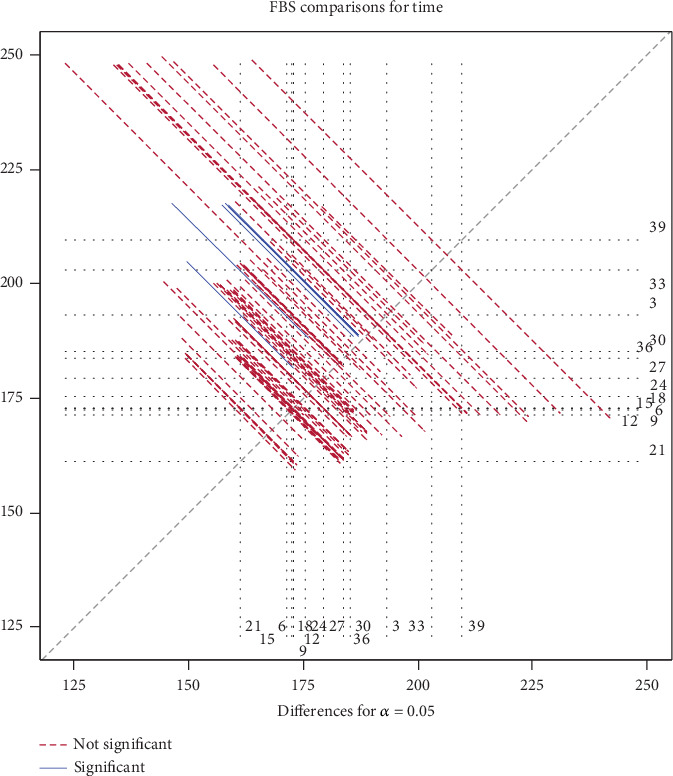
Comparison of mean fasting blood sugar over time in diabetes patients.

Furthermore, the FBS levels' initial decline, midphase stability, and late-phase increase follow a temporal pattern that is in line with clinical expectations for long-term diabetes management. These findings and the visual patterns shown in Figure 3 highlight the need for longitudinal models to take time-varying effects into consideration and the inadequacy of baseline measurements in capturing the evolution of glycemic control.

The study's central hypothesis that FBS levels are not constant but rather fluctuate greatly over the course of the follow-up period, requiring longitudinal analytical approaches, is supported, in conclusion, by the statistically significant differences across visit times.

The deviance information criterion (DIC) was used to select the best fitting model for the dataset. The random coefficient model with time-varying covariates provided the best fit, exhibiting the smallest −2 log likelihood value of 9698.5, as shown in Table 7. This indicates that the fixed variables vary at both the patient level and the individual visit time level for the FBS levels of diabetic patients.

The parameter estimations for the random coefficient time-varying covariate model applied to the FBS levels of diabetes patients are presented in Table 8. The results include estimates of parameters, standard errors, *p* values, and 95% confidence intervals for each parameter.

To predict the response variable, several explanatory covariates were utilized, including age group, visiting time, hypertension status, pulse rate, creatinine levels, cholesterol levels, LDL, HDL, triglycerides, and baseline FBS. All of these variables were found to be significantly associated with FBS levels in diabetes patients at a significance level of 0.25. These covariates were key determinants in assessing patients' FBS levels over time.

In the random coefficient time-varying covariate model (see Supporting Information for the full SAS code), pulse rate, HDL, and baseline FBS were shown to be statistically significant, with associations at a 5% significance level. Notably, the parameter estimation for pulse rate was 1.3108, with a 95% confidence interval of (0.4496, 2.1719), indicating a significant relationship with the FBS levels of diabetes patients (*p* value = 0.0029).

**Figure 4:**
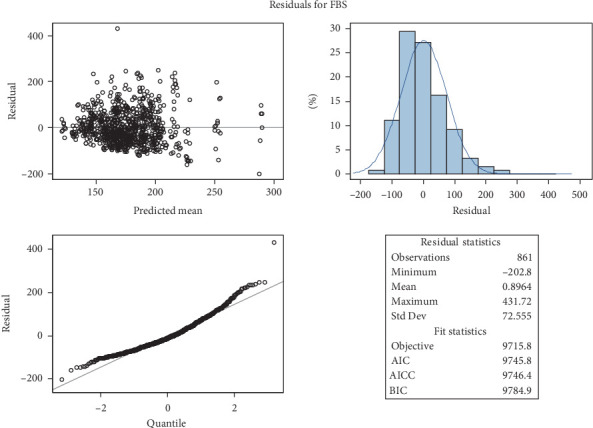
Residual plot for model diagnostics of fasting blood sugar prediction in diabetes patients.

**Figure 5:**
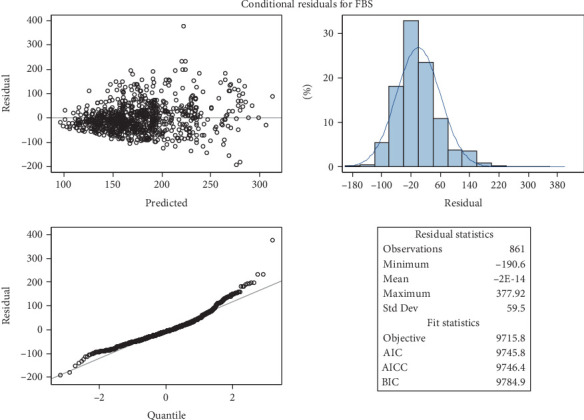
Conditional residual plots of predicted versus residuals for fasting blood sugar levels in diabetes patients.

Diagnostic plots used to assess the assumptions of the multilevel random coefficient model used in this investigation are shown in Figures 4 and 5. In particular, the residual plot for FBS levels in diabetic patients is shown in Figure 4, and the conditional plot of predicted values versus residuals is shown in Figure 5. The model assumptions, especially those pertaining to the residual distribution and the suitability of the model fit, must be validated using these plots. These figures have a crucial methodological function in determining whether the applied model is suitable for the data structure, even though their current appearance may resemble default software outputs (see Supporting Information for the full SAS code).

It is crucial to remember that the multilevel random coefficient model does not require that the outcome variable or time-varying covariates be normal. The main presumptions concern the residuals and random effects' approximate normality. The central limit theorem confirms that the model is robust against moderate departures from normalcy, especially considering the large sample size (*n* = 861).

A rather random distribution of residuals around zero is depicted in Figure 4 (the residual plot), indicating homoscedasticity (constant variance) and no significant deviations from the independence or linearity assumptions. The model's suitability is further supported by Figure 5's conditional residual plot, which shows no discernible pattern between residuals and predicted values. This suggests that the model's predictions are objective and that the residuals are roughly normally distributed (see Supporting Information for the full SAS code).

A rather random distribution of residuals around zero is depicted in Figure 4 (the residual plot), indicating homoscedasticity (constant variance) and no significant deviations from the independence or linearity assumptions. The model's suitability is further supported by Figure 5's conditional residual plot, which shows no discernible pattern between residuals and predicted values. This suggests that the model's predictions are unbiased across the range of observed values and that the residuals are roughly normally distributed.

When combined, these diagnostic plots demonstrate that the assumptions of the multilevel model are sufficiently satisfied, supporting the validity of the model's estimates and conclusions about how time and covariates affect FBS levels. To prevent the misunderstanding that these figures are raw software outputs, it is essential to properly interpret and methodologically justify them.

## 4. Discussions

This study investigated the longitudinal trajectories of FBS among adult diabetic patients at JUSH by applying a multilevel random coefficient model with time-varying covariates. The data consisted of repeated measurements (Level 1) of FBS across up to 13 visits nested within 861 individual patients (Level 2). This modeling approach enhances understanding of both within-patient (Level 1) dynamics over time and between-patient (Level 2) heterogeneity in glycemic control in a real-world Ethiopian clinical context.

### 4.1. Interpretation of Key Findings

The model identified three significant predictors of FBS change over time:

• *Pulse rate* was positively associated with FBS (*p* = 0.0029), suggesting that a higher resting heart rate may signal poorer glycemic control. This aligns with broader evidence linking elevated heart rate with increased risk of Type 2 diabetes [25, 26] and may reflect autonomic imbalance or heightened cardiovascular stress in diabetic individuals. The pulse rate at diagnosis was particularly predictive: For each unit increase in pulse rate, FBS increased by 3.7 times (*β* = 1.3108, 95% CI: 0.4496–2.1719). This finding is consistent with prior studies showing heart rate as an independent predictor of diabetes incidence and mortality [27, 28]. However, resting heart rate's utility as a screening tool for undiagnosed diabetes in rural populations is limited [25].
• *HDL* levels were unexpectedly positively associated with FBS (*p* = 0.0436). Although HDL is generally considered protective in metabolic disorders [29, 30], such counterintuitive results may stem from confounding by medication or nutritional changes. Swiss research has linked low HDL levels with a higher risk of Type 2 diabetes and other diseases [30]. In diabetes patients, HDL function may be impaired by oxidative modification and glycation, converting HDL into proinflammatory agents [29]. Other studies also highlight HDL's complex association with diabetes [31–33]. Further research is needed to clarify this relationship in the Ethiopian context.
• *Baseline FBS* was a strong predictor (*p* < 0.0001), underscoring the prognostic importance of initial glycemic status for subsequent trends. The risk associated with baseline FBS varied within and between individuals, contributing to increased diabetes progression risk. This finding echoes longitudinal studies emphasizing baseline glycemia as a key determinant of future glucose control.

Other variables including creatinine, LDL, total cholesterol, triglycerides (approaching significance, *p* = 0.0980), hypertension status, and age group did not achieve statistical significance. While older age showed a nonsignificant trend toward higher FBS, this aligns with evidence of age-related declines in insulin sensitivity, which may require larger samples or stratified analysis to detect (e.g., Felege Hiwot and Debre Markos referral hospitals' study).

Interestingly, visit time itself lacked a significant fixed effect (*p* = 0.8887), suggesting that individual-level variations (random effects) outweighed a uniform time trend. Nevertheless, visual trends (Figure 2) and time-specific comparisons (Table 6) demonstrated meaningful fluctuations in FBS across visits, validating the utility of a random coefficient framework.

### 4.2. Context Within Existing Literature

Most Ethiopian studies exploring FBS have relied on cross-sectional data, limiting insight into temporal dynamics. This study's use of longitudinal multilevel modeling offers methodological advantages by enabling the following:

1. Within-subject (intraindividual) change analysis,
2. Between-subject variability modeling through random effects, and
3. Incorporation of time-varying predictors, which are often overlooked in prior work.

For instance, Moges et al. [34] employed longitudinal models to examine FBS progression but did not model time-varying covariates or individual-level variation. Studies in the Tigray Region and Northwest Ethiopia similarly focused on broad time trends or joint modeling without such a comprehensive multilevel structure (Abuhay et al., [35]; joint model of retinopathy and FBS). Our study adds to this emerging literature by explicitly modeling random intercepts and slopes with time-varying predictors in a substantial patient cohort.

### 4.3. Implications for Clinical Practice and Policy

Together, these findings advocate for the following:

1. Comprehensive cardiovascular monitoring, given the association of pulse rate with FBS trends,
2. Early and ongoing assessment of glycemic control, as indicated by baseline FBS's predictive power, and
3. Individualized care plans, recognizing high heterogeneity in patient responses over time.

This modeling strategy can be applied in resource-constrained settings across Ethiopia to analyze routinely collected clinical data, thereby informing precision medicine and data-driven policy.

### 4.4. Main Contribution of the Study

In summary, the study's principal contribution lies in its rigorous application of a random coefficient time-varying covariate model to a large longitudinal dataset in Ethiopia. This approach establishes a new benchmark for methodological sophistication in regional diabetes research, enabling simultaneous investigation of intra individual trajectories and interindividual differences.

## 5. Conclusion

The main purpose of this study was to identify potential covariates associated with FBS levels in diabetes patients under follow-up care at JUSH, Jimma, Ethiopia. The study utilized a series of repeated measurements over time, with the lowest level observations nested within individual patients. The findings revealed that 70.2% of the variation in FBS levels among diabetic patients occurred at this lowest level. Thus, the results from the multilevel random coefficient time-varying covariate model indicate that pulse rate, HDL levels, and baseline FBS levels were significant predictors of FBS levels in diabetes patients at a 5% significance level. For instance, for every one beat per minute increase in pulse rate, FBS levels increased by 1.31 mg/dL. Similarly, FBS levels increased by 0.35 mg/dL for each milligrams per deciliter change in HDL levels. Moreover, FBS levels changed by 0.02 mg/dL with each visit when baseline FBS levels changed by 0.02 mg/dL. Therefore, it is crucial to provide special care to patients during follow-up visits, and nursing interventions should focus on reducing stress, avoiding tobacco products, and promoting exercise to improve both pulse rate and FBS levels.

## Figures and Tables

**Table 1:** Study covariates.

**S. no.**	**Variables**	**Categories**
1.	Gender	0 = female, 1 = male
2.	Age group	0 = < 20, 1 = 20–44, 2 = 45–64, 3 = ≥ 65
3.	Baseline fasting blood sugar	Continuous
4.	Level of glucose	Continuous
5.	Level of triglyceride	Continuous
6.	Level of high-density lipoprotein	Continuous
7.	Cholesterol level	Continuous
8.	Level of low-density lipoprotein	Continuous
9.	Level of urea	Continuous
10.	Level of creatinine blood test	Continuous
11.	Residence	0 = rural, 1 = urban
12.	Pulse rate	Continuous
13.	Baseline systolic blood pressure	Continuous
14.	Baseline diastolic blood pressure	Continuous
15.	Hypertension status	0 = no, 1 = yes
16.	Complication status	0 = no, 1 = yes
17.	Types of complication happened	0 = no complication, 1 = short-term complication, 2 = long-term complication

**Table 2:** Descriptive statistics for categorical covariates.

**Variables**	**Levels**	**Fasting blood sugar**				
		**Mean**	**SD**	**Minimum**	**Maximum**	**Count (%)**
Gender	Female	170.18	71.34	65.00	422.00	365 (42.4)
	Male	182.51	78.92	62.00	600.00	496 (57.6)

Hypertension	No	182.59	81.22	65.00	456.00	386 (44.8)
	Yes	172.97	71.28	62.00	600.00	475 (55.2)

Residence	Rural	178.94	80.62	64.00	600.00	454 (52.7)
	Urban	175.43	70.54	62.00	422.00	407 (47.3)

Complication	No	179.84	76.22	62.00	600.00	512 (59.5)
	Yes	173.52	75.62	64.00	415.00	349 (40.5)

Types of complications	No	179.50	75.64	62.00	600.00	529 (61.4)
	Short term	177.69	76.56	64.00	385.00	174 (20.2)
	Long term	169.39	76.55	65.00	415.00	158 (18.4)

Age group	Less than 20	281.83	110.75	85.00	385.00	6 (0.7)
	20–44	186.60	79.22	65.00	600.00	198 (23)
	45–64	165.11	67.57	64.00	451.00	439 (51)
	≥ 65	190.45	82.70	62.00	448.00	218 (25.3)

**Table 3:** Summary of fasting blood sugar levels at different time points.

Time	3	6	9	12	15	18	21	24	27	30	33	36	39
*n*	98	98	96	91	84	79	69	64	58	50	40	30	4
Mean	193.8	174.1	173.3	173.0	171.5	171.9	161.9	175.9	177.1	181.3	203.8	182.5	209.8
SD	76.4	78.14	84.58	84.46	69.48	64.32	66.85	73.12	77.32	68.82	85.27	65.56	135.90

**Table 4:** Descriptive statistics for continues covariates over the visiting time of the patients' during the follow-up period.

**Visiting time of patients**														
**Variables**	**Statistic**	**1st**	**2nd**	**3rd**	**4th**	**5th**	**6th**	**7th**	**8th**	**9th**	**10th**	**11th**	**12th**	**13th**
Pulse rate	Mean	85.08	85.16	85.34	85.54	86.11	86.08	86.23	86.17	85.78	85.72	86.63	86.17	83.75
	SD	10.18	10.11	10.13	10.29	10.29	10.22	10.55	10.81	10.72	10.13	9.63	10.29	5.68
BSBP	Mean	130	130	130	130	130	130	130	130	131	131	133	133	145
	SD	20	19	19	19	18	18	19	19	19	20	20	22	26
BDBP	Mean	80	80	80	80	81	81	81	81	81	82	82	81	80
	SD	10	10	10	10	9	9	10	10	10	10	10	11	8
Weight	Mean	68	68	69	69	69	69	70	70	69	69	69	69	71
	SD	12	12	12	11	12	12	11	12	12	12	12	13	8
Creatinine level	Mean	1.159	1.161	1.168	1.149	1.134	1.143	1.171	1.138	1.164	1.136	1.174	1.294	1.253
	SD	0.596	0.594	0.598	0.591	0.593	0.608	0.610	0.548	0.555	0.494	0.525	0.518	0.272
Level of urea	Mean	57.06	56.64	57.17	56.16	53.57	53.64	55.34	55.44	58.10	58.25	62.74	72.10	61.00
	SD	40.18	40.24	40.49	40.05	38.48	37.96	38.61	39.29	39.99	41.90	44.79	47.42	26.77
Cholesterol level	Mean	146.95	146.99	148.87	146.56	144.95	145.16	151.16	150.38	152.54	153.19	160.80	159.96	160.50
	SD	50.86	50.83	49.53	48.87	47.50	47.91	45.44	44.94	45.61	44.25	44.19	43.22	34.04
LDL	Mean	95.64	95.68	96.31	95.36	93.18	92.55	96.00	96.87	98.64	96.98	98.57	104.10	113.25
	SD	41.44	41.37	41.15	40.84	41.72	42.50	42.60	43.10	42.89	43.43	43.03	41.21	20.56
HDL	Mean	64.68	64.56	64.63	64.59	65.46	65.07	66.34	67.45	68.42	67.25	69.75	72.83	71.75
	SD	25.36	25.44	25.61	25.42	25.41	25.88	26.76	26.96	26.97	27.07	28.44	29.25	24.80
Level of triglyceride	Mean	152.50	152.90	154.01	148.72	146.85	147.81	150.91	151.58	156.49	154.47	158.49	170.02	175.00
	SD	67.65	67.59	67.78	64.66	63.74	64.69	67.43	68.91	70.22	71.98	65.82	67.45	81.99
Level of glucose	Mean	258.77	257.88	258.46	258.61	261.72	258.08	261.12	261.92	253.70	247.56	256.33	255.94	275.75
	SD	83.94	84.67	85.43	87.57	88.93	90.06	93.29	95.09	87.00	85.24	84.46	87.11	84.54
Baseline FBS	Mean	260.60	260.57	260.58	261.58	267.82	267.85	264.55	267.45	269.48	267.90	275.85	275.70	279.75
	SD	84.52	85.67	85.32	86.47	86.30	88.54	86.43	88.15	84.73	88.87	90.68	96.46	55.48

**Table 5:** Intercept-only model estimates.

**Model**	**Effects**	**Estimate**	**Sd. Err**	**Pr** > |**t**|	**Lower**	**Upper**
Solution for fixed effects	Intercept	176.05	4.7932	< 0.0001	166.54	185.56
Covariance parameter estimates	Intercept (id)	1739.84	330.19	< 0.0001	1238.48	2623.29
	Residual	4096.34	210.33	< 0.0001	3713.45	4541.97

*Note:* −2 log likelihood = 9753.5. AIC = 9759.5. BIC = 9767.3. Null model likelihood ratio test is significant with chi-square 146.45 and *p* value < 0.0001.

**Table 6:** Results of time least squares mean comparison for visiting time.

**Time least squares means**					
**Time**	**Estimate**	**Standard error**	**DF**	**t** ** alue**	**Pr** > |**t**|
3	192.90	6.4355	749	29.97	< 0.0001
6	174.23	6.4639	749	26.95	< 0.0001
9	173.39	6.5623	749	26.42	< 0.0001
12	172.88	6.7926	749	25.45	< 0.0001
15	167.57	7.1309	749	23.50	< 0.0001
18	167.70	7.3835	749	22.71	< 0.0001
21	162.10	7.9497	749	20.39	< 0.0001
24	175.77	8.2733	749	21.25	< 0.0001
27	176.26	8.7078	749	20.24	< 0.0001
30	179.69	9.3949	749	19.13	< 0.0001
33	198.19	10.5162	749	18.85	< 0.0001
36	172.94	12.1438	749	14.24	< 0.0001
39	181.55	33.1324	749	5.48	< 0.0001

**Table 7:** Deviance information criteria for multilevel analysis.

**Models**	**−2 log likelihood**
Intercept-only model	9753.5
Random intercept time-varying covariate model	9703.8
Random intercept time-invariant covariate model	9706.7
Random coefficient time-invariant covariate model	9720.7
Random coefficient time-varying covariate model	9698.5

**Table 8:** Parameter estimation of random coefficient time-varying covariate model.

**Solution for fixed effects**						
**Effects**	**Levels**	**Estimate**	**SdError**	**Pr** > |**t**|	**Lower**	**Upper**
Intercept		69.5161	53.2439	0.1950	−36.2463	175.28
Vising time	Continues	4.6521	33.2242	0.8887	−60.5719	69.8761
Age group (≥ 65)	< 20	72.4382	57.1487	0.2054	−39.7532	184.63
	20–44	4.9152	12.0991	0.6847	−18.8372	28.6676
	45–64	−18.9238	10.1242	0.0620	−38.7990	0.9515
Hypertension status (yes)	No	1.4208	8.2525	0.8634	−14.7800	17.6217
Pulse rate level	Continues	1.3108	0.4387	0.0029	0.4496	2.1719
Creatinine level	Continues	−10.6877	8.0244	0.1833	−26.4408	5.0653
Cholesterol level	Continues	−0.03747	0.09541	0.6946	−0.2248	0.1498
LDL	Continues	−0.1377	0.1161	0.2357	−0.3656	0.09010
HDL	Continues	0.3483	0.1723	0.0436	0.01005	0.6866
Triglyceride level	Continues	0.1193	0.07204	0.0980	−0.02208	0.2608
Baseline FBS	Continues	0.01633	0.003593	< 0.0001	0.01107	0.02650
−2 log likelihood	= 9698.5	.	AIC = 9748.5	BIC = 9813.7		*N* = 861

## Data Availability

The data that support the findings of this study are available on request from the corresponding author. The data are not publicly available due to privacy or ethical restrictions.

## Nomenclature

SAS: Statistical Analysis System
ART: antiretroviral therapy
WHO: World Health Organization
FBS: fasting blood sugar
BMI: body mass index
WC: circumference
IDF: International Diabetes Federation
LMM: linear mixed model
LDL: low-density lipoprotein
HDL: high-density lipoprotein
ICC: intraclass correlation coefficient
VPC: variance partition coefficient
AIC: Akaike information criteria
BIC: Bayesian information criteria
DIC: deviance information criterion

## Supporting Information

Supporting Information Additional supporting information can be found online in the Supporting Information section. Additional supporting information is provided in the Supporting Information. This section includes the SAS code used for the statistical analyses conducted in this study. The code covers procedures for sorting data, plotting individual fasting blood sugar levels over time, and performing the mixed-effects model used to analyze the longitudinal data.
