# Supplementary material for: Longitudinal Study on the Progression of Diabetes Mellitus Patients at Jimma University Specialized Hospital, Ethiopia
Source: J Diabetes Res. 2025 Dec 3;2025:7035393. doi: 10.1155/jdr/7035393 (PMC12695411; doi:10.1155/jdr/7035393)
Supplement: Supporting Information — Additional supporting information can be found online in the Supporting Information section. Additional supporting information is provided in the Supporting Information. This section includes the SAS code used for the statistical analyses conducted in this study. The code covers procedures for sorting data, plotting individual fasting blood sugar levels over time, and performing the mixed-effects model used to analyze the longitudinal data. [file 7035393.f1.docx]

**Appendix**

SAS Codes for Data Analysis

**proc** **sort** data=dm;

by time;

**run**;

**proc** **gplot** data=dm;

plot FBS*time =id/ nolegend haxis= **0** to **39** by **3** vaxis= **50** to **400** by **20** ;

symbol v=none repeat=**861** i=joint;

label time ='Visit Time' FBS='Fasting Blood Sugar levels ';

title 'Profile Plot for Individual Fasting Blood Sugar levels';

**run**;

**proc** **mixed** data=dm noclprint covtest cl method=ml;

class id gender HT age_group;

M5: model FBS=time age_group HT PR creatinine cholestrol_level LDL HDL Triglceride/s cl residual;

random intercept time /sub=id;

**run**;

**proc** **sgplot** data=dm;

vline time/response =FBS stat= mean limitstat=stderr;

yaxis label='mean of FBS';

**run**;
